# Soil aggregate stability and its influencing factors in *Picea Schrenkiana var. Tianschanica* forests of the Central Tianshan Mountains

**DOI:** 10.1371/journal.pone.0359791

**Published:** 2026-10-09

**Authors:** Yuwen Chen, Weixia Wang, Shanchao Zhao, Xuansheng Huang, Haodong Yang, Yubo Sun, Xiaoli Ma, Weiye Du, DongLin Zhang, Zhimin Xi

**Affiliations:** 1 College of Forestry and Landscape Architecture, Xinjiang Agricultural University, Urumqi, Xinjiang, China; 2 Xinjiang Department of Education Key Laboratory of Arid Land Forestry Ecology and Industrial Technology, Urumqi, Xinjiang, China; 3 Natural Forest Protection Center of Uygur Autonomous Region of Xinjiang, Urumqi, Xinjiang, China; Zhejiang Agriculture and Forestry University: Zhejiang A and F University, CHINA

## Abstract

Soil aggregate stability (SAS) is a key indicator of soil structural quality that underpins erosion resistance, water regulation, nutrient retention, and ecosystem functioning. We examined how topography and forest stand structure were associated with soil aggregate-size distribution and stability in Picea schrenkiana var. tianschanica forests in the central Tianshan Mountains, China. Water-stable aggregates and soil physicochemical properties were measured in 188 plots at two soil depths (0–20 and 20–60 cm). Linear mixed-effects models (MEMs), redundancy analysis (RDA), and structural equation modeling (SEM) were used to evaluate complementary univariate, multivariate, and hypothesized pathway-level relationships. Fine aggregates (0.25–0.053 mm) constituted the largest fraction (21.2–49.3%). Descriptively, aggregate stability tended to be higher on 25–35° slopes, in valley and downslope positions, on sunny aspects, and at elevations of 2300–2700 m. After accounting for plot-level dependence and sampling year, exposure and stand age were significant predictors of MWD, whereas slope, slope position, altitude, and canopy density were not. SOM showed the strongest positive association with SAS in the SEM (standardized β = 0.49), whereas bulk density showed a negative association (β = −0.41). The combined analyses indicate that terrain and stand structure are associated with SAS partly through variation in SOM, soil water content, and bulk density; because the study was observational, the modeled paths should not be interpreted as proof of causation. These findings identify soil organic matter retention and avoidance of compaction as priorities for maintaining soil structural resilience in high-altitude spruce forests.

## Introduction

Soil is a foundational component of terrestrial ecosystems and provides essential ecosystem services. Soil aggregates, the basic structural units of soil, influence porosity, water retention, permeability, and resistance to erosion [1–3]. Stable aggregates enhance water and nutrient retention and help maintain soil fertility and productivity [4]. Accordingly, soil aggregate stability (SAS) is widely used as an integrative indicator of soil structural quality and soil health, particularly in forest ecosystems where long-term productivity and resilience depend on stable pore networks and erosion resistance [5–7].

The formation and stability of soil aggregates are governed by intrinsic soil properties and external environmental drivers. Soil texture and clay content influence aggregation through particle-size distribution and physicochemical bonding [8–10]. Soil organic matter promotes aggregate formation and persistence through binding and cementing mechanisms, while soil moisture regulates microbial activity and organic-matter turnover [11]. In forests, vegetation contributes through roots, litter inputs, and rhizosphere interactions [12]. Topographic factors such as aspect, slope position, and elevation alter microclimate and hydrological pathways and may therefore influence the spatial distribution of SAS [13,14].

Mountain forest ecosystems are especially sensitive to these interacting controls due to steep environmental gradients and strong spatial heterogeneity. Variations in topography and stand structure can jointly regulate soil moisture regimes, organic matter inputs, and biological activity, leading to complex spatial patterns of SAS. Recent studies increasingly highlight that slope position and related erosion/deposition processes can strongly structure aggregate stability patterns at landscape scales, reinforcing the need to account for terrain-mediated redistribution processes when interpreting SAS [15,16].

The natural forests of Picea schrenkiana var. tianschanica in the Central Tianshan Mountains of Xinjiang represent a critical ecological barrier for water conservation, biodiversity maintenance, and carbon sequestration in arid and semi-arid regions of Northwest China [17,18]. These forests develop under pronounced topographic gradients and distinct stand structures, which strongly influence soil structure development and nutrient dynamics [19,20]. Despite substantial progress in understanding soil structural dynamics, existing studies on forest soil aggregation have largely examined single drivers (e.g., topography or vegetation) in isolation, or have relied on bivariate correlations that fail to separate direct effects from indirect pathways mediated by soil properties. Consequently, for high-altitude Picea schrenkiana var. tianschanica forests, evidence remains scarce regarding how topography and stand structure jointly regulate SAS across soil depths and through which specific pathways (e.g., organic matter, moisture, bulk density) these controls operate. Furthermore, recent stand-development work highlights that forest development can systematically shift aggregate-size distribution and strengthen aggregate-associated carbon protection, emphasizing the necessity of explicitly integrating stand age and structural trajectories into stability assessments [21].

Accordingly, we tested the following hypotheses: (H1) SAS is higher in terrain settings that favor organic matter accumulation and moderate hydrothermal conditions (e.g., valley positions, gentle slopes, and sunny aspects), primarily via increased soil organic matter and soil moisture; (H2) younger stands and higher canopy closure enhance SAS by increasing litter inputs and root-related binding processes, especially in surface soils; and (H3) the effects of topography and stand structure on SAS are predominantly indirect, operating through soil organic matter, moisture, and bulk density rather than through direct pathways alone. To evaluate these hypotheses, we aimed to (1) quantify SAS and aggregate-size distribution across gradients of slope, aspect, position, and elevation; (2) assess the influence of stand age and canopy closure on SAS across two soil depths; and (3) disentangle direct and indirect pathways linking terrain and stand attributes to SAS using an integrated analytical framework.

## Materials and methods

### Study area

The study was conducted at the Tianger Campus experimental forest of Xinjiang Agricultural University (approximately 110 km southwest of Urumqi), located in the central part of the northern foothills of the Tianshan Mountains, Xinjiang, China. The area lies in the upper reaches of the Toutun River watershed, characterized by mountainous terrain with elevations ranging from 1700 to 2700 m above sea level and slope gradients between 10° and 40°. The region experiences a temperate continental climate with a mean annual precipitation of approximately 600 mm, a mean annual temperature of about 3 °C, and an average frost-free period of 140 days. The forest vegetation is dominated by natural pure stands of *Picea schrenkiana var. tianschanica*, with understory herbaceous species including Aegopodium alpestre, Galium boreale, and Iris ruthenica. Soils are classified as gray-brown forest soils. This study did not require special permits, as the sampling was conducted within the university’s experimental forest grounds and involved no direct disturbance to protected flora or fauna.

### Experimental design and plot selection

Field sampling was conducted during June–August in 2021–2023. A stratified design was used to represent variation in topography and stand structure. Each plot was sampled in one field season; sampling year was therefore treated as a between-plot design factor rather than as a repeated measure.

Stratification: Slope was divided into four classes (<15°, 15–25°, 25–35°, > 35°); slope position into five categories (valley, lower slope, middle slope, upper slope, ridge); slope aspect into four categories (sunny, semi-sunny, shady, semi-shady); and elevation into five ranges (between 1700 m and 2700 m at 200 m intervals). Stand age was classified via tree-ring methods into young (<60 years), middle-aged (61–100 years), near-mature (101–120 years), and mature (121–160 years). Canopy closure was visually estimated and categorized into low (0.20–0.39), medium (0.40–0.60), and high (>0.60).

Plot setup and soil sampling: We established 188 plots (30 m × 30 m), each assigned a unique Plot ID. Within each plot, soil was collected at three spatially distributed points after the litter layer had been removed. At each point, samples were taken from 0–20 cm and 20–60 cm. The three samples from a given depth were homogenized to form one composite sample per plot and depth, yielding at most 376 plot-depth observations before exclusion of missing records. Approximately 500 g of each composite sample was retained by quartering. One undisturbed 100-cm^3^ core was collected at each plot-depth combination for bulk-density measurement. Volumetric soil water content (SWC) was measured in situ with a calibrated LD-S portable soil-moisture meter (Laiende Instrument Co., China). The plot, not the within-plot sampling point, was the independent sampling unit.

### Soil physicochemical analyses

Composite samples were air-dried in a shaded, ventilated room, gently disaggregated without crushing intact mineral particles, and passed through a 2-mm sieve. Soil organic matter (SOM) was measured by potassium dichromate oxidation; total nitrogen (TN) by Kjeldahl digestion; alkaline hydrolyzable nitrogen (ASN) by alkaline hydrolysis diffusion; available phosphorus (A-P) by molybdenum–antimony colorimetry; and available potassium (A-K) by ammonium acetate extraction followed by flame photometry. Soil pH was measured potentiometrically in a 1:2.5 soil:water suspension. Total porosity (TP) was calculated as TP (%) = [1 − BD/PD] × 100, where BD is measured bulk density and PD is particle density.

### Protocol for soil aggregate stability (dry and wet sieving)

The same aggregate-size boundaries were used for dry and wet sieving: > 2.0, 2.0–0.5, 0.5–0.25, 0.25–0.053, and <0.053 mm. Mechanically stable fractions were obtained by dry sieving; water-stable fractions were obtained by wet sieving. All masses used in the calculations were oven-dry-equivalent masses.

Dry sieving: A 100-g air-dried subsample in which natural aggregates had been preserved was placed on nested 2.0-, 0.5-, 0.25-, and 0.053-mm sieves. The stack was shaken vertically for 5 min. Material retained on each sieve and passing the 0.053-mm sieve was collected and weighed. The mass fraction of each class was calculated relative to the recovered total mass.

Wet sieving: A 50-g subsample was reconstructed in the same proportions as the dry-sieved size distribution. It was placed in a 1000-mL cylinder, pre-moistened, and immersed for 10 min. The saturated sample was transferred to the nested sieve set while submerged in water and oscillated vertically through a 3-cm stroke at 30 cycles min − 1 for 5 min. Material retained in each size class and passing the smallest sieve was transferred quantitatively to pre-weighed aluminum pans, oven-dried at 105 °C to constant mass, cooled, and weighed.

Soil aggregate stability (SAS) was quantified using six standard indices. Equations Eq. (1)–(6) are fully defined as follows:

1. Proportion of water-stable macroaggregates (R_>0.25_):

$$\begin{array}{c} R_{>0.25}=\frac{M_{>0.25}}{M_{T}}\times 100\% \end{array}$$ (1)

Here, M_>0.25_ is the oven-dry mass of water-stable aggregates >0.25 mm (g), and M_T_ is the recovered total oven-dry mass after wet sieving (g).

2. Mean Weight Diameter (MWD):

$$\begin{array}{c} MWD=\sum_{i}^{n}\overset{―}{X_{l}}W_{i} \end{array}$$ (2)

Where $\overset{―}{X_{i}}$ is the mean diameter of the i-th size fraction (mm), $W_{i}$ is the mass proportion of the i-th size fraction, and n is the number of size fractions.

3. Geometric Mean Diameter (GMD):

$$\begin{array}{c} GMD=exp\left(\frac{\sum W_{i\left(Inx_{i}\right)}}{\sum W_{i}}\right) \end{array}$$ (3)

4. Fractal Dimension (D):

$$\begin{array}{c} (3-D)lg\left(\frac{\overset{―}{d_{i}}}{\overset{―}{d_{max}}}\right)=lg\left[\frac{W(\delta <\overset{―}{d_{i}})}{W_{\text{0}}}\right] \end{array}$$ (4)

Where 3−D is the slope of the fitted equation,D is the fractal dimension of soil particles,$\overset{―}{d_{\text{i}}}$ represents the average of the two neighboring particle sizes,$\overset{―}{{\text{d}}_{\text{max}}}$ represents the average diameter of the largest-sized soil particle,$W\text{(}\delta <\overset{―}{d_{i}}\text{)}$ is the cumulative soil mass of the aggregates smaller than $\overset{―}{d_{\text{i}}}$ and $W_{\text{0}}$ indicates the sum of soil mass for each particle size of the soil.

5. Percentage of Aggregate Destruction (PAD):

$$\begin{array}{c} PAD=\frac{\left(DR_{0.25}-WR_{0.25}\right)}{DR_{0.25}}\times 100\% \end{array}$$ (5)

Where $DR_{>0.25}$ is the mass of mechanically stable aggregates >0.25 mm obtained from dry sieving, and $WR_{>0.25}$ is the mass of water-stable aggregates >0.25 mm obtained from wet sieving.

6. Unstable Aggregate Index (E_LT)_:

$$\begin{array}{c} E_{LT}=\frac{\left(W_{T}-WR_{0.25}\right)}{W_{T}}\times 100\text{\textbackslash{}\%} \end{array}$$ (6)

### Statistical analyses and modeling framework

Analyses were conducted in R 4.2.2. The number of observations used in each analysis was recorded after listwise deletion of records with missing values (<1%). Continuous variables were inspected graphically and with model residual diagnostics; transformations were applied when needed to improve residual normality and variance homogeneity. For descriptive comparisons among the categories shown in Figs 1–6, estimated marginal means from the mixed-effects models were compared with Tukey adjustment for multiple testing at α = 0.05. Effect estimates, confidence intervals, and exact P values were emphasized rather than relying only on significance labels.

**Fig 1:**
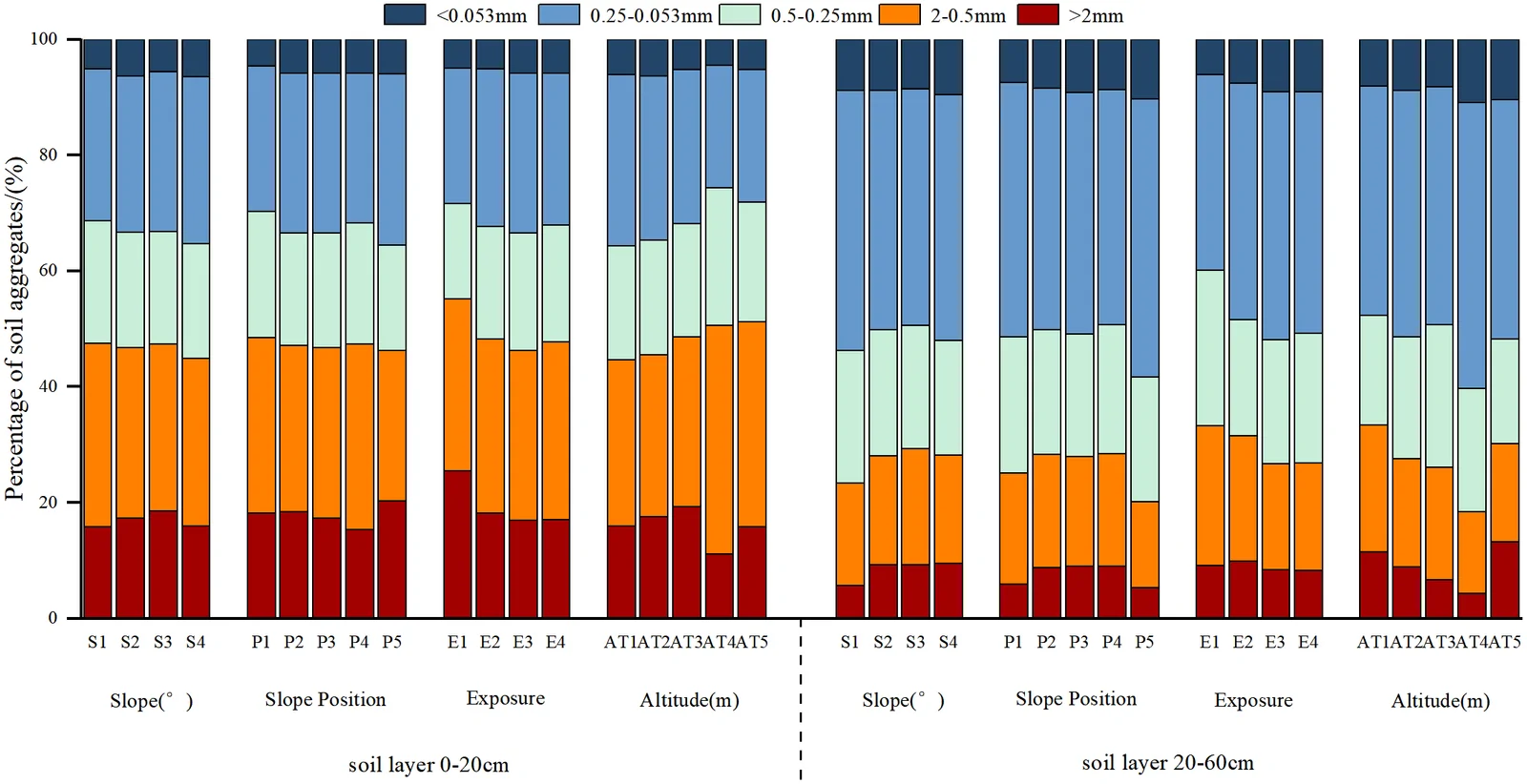
Soil water stability aggregate composition at different depths under different terrain conditions. Note: Some letters in the figure indicate: S1: slope <15°; S2: slope 15°-25°: S3: slope 25°-35°; S4: slope >35°; P1: valley; P2: uphill; P3: middle slope; P4: downhill; P5: ridge; E1: sunny slope; E2: semi- sunny slope; E3: shady slope; E4: semi-shady slope; AT1: altitude 1700-1900m; AT2: altitude 1900-2100m; AT3: altitude 2100-2300m; AT4: altitude 2300-2500m; AT5: altitude 2500-2700m.

**Fig 2:**
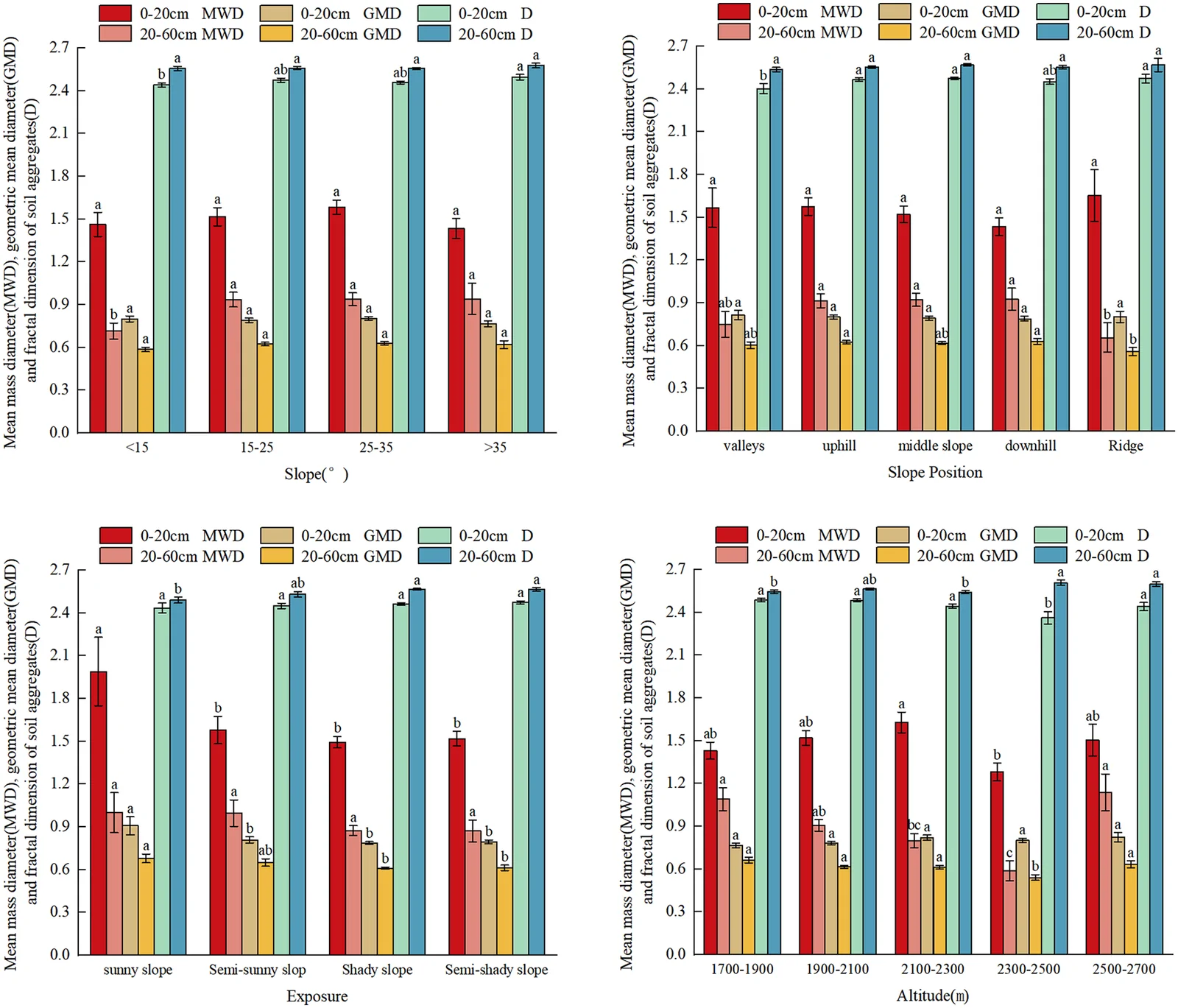
Mean weight diameter (MWD), Geometric mean Diameter (GMD) and fractal dimension (D) of water-stable aggregates under different topographic conditions. Note: Different lowercase letters indicate significant differences between different terrain conditions in the same soil layer (P < 0.05).

**Fig 3:**
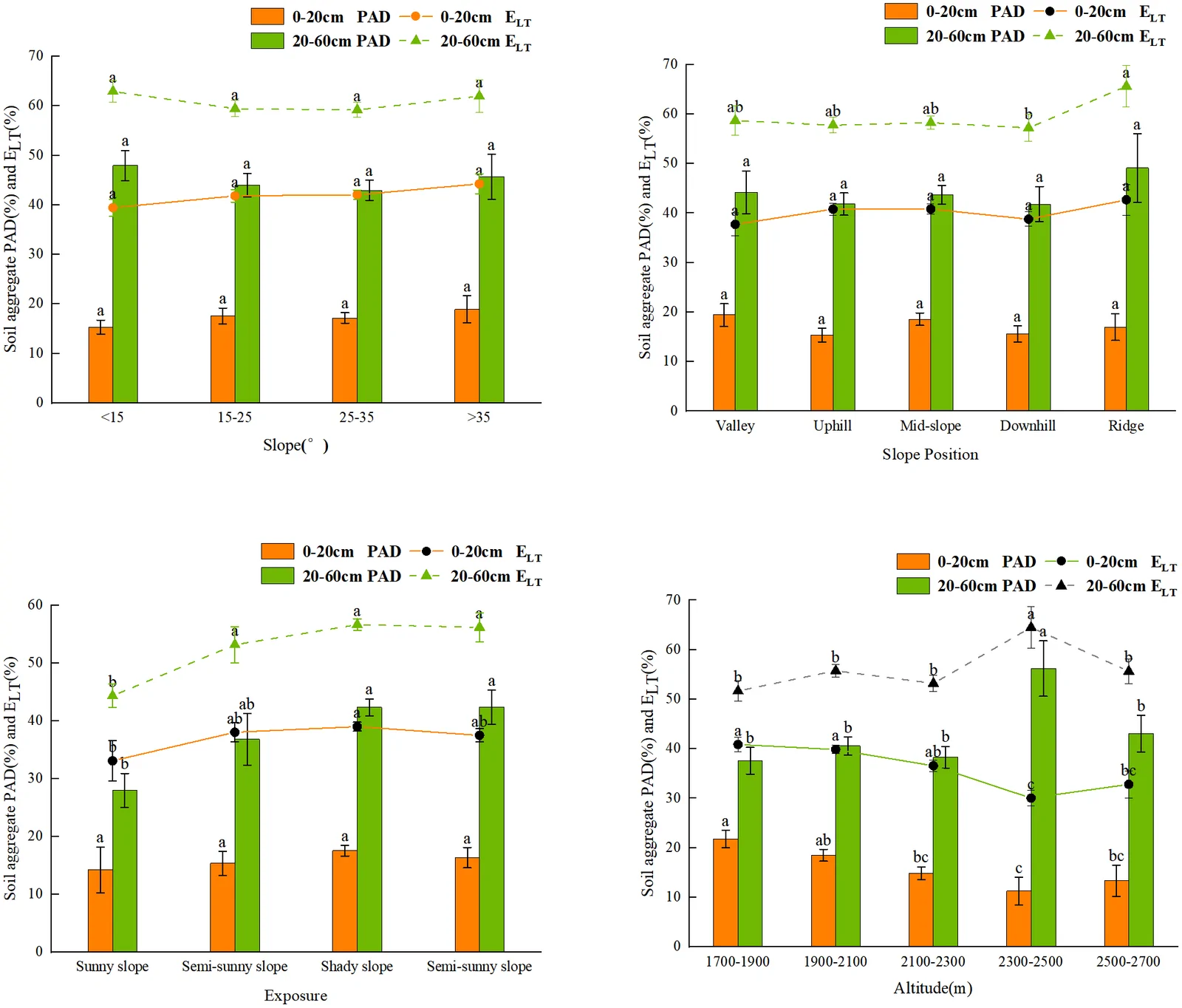
Soil aggregate failure rate (PAD) and unstable aggregate index (E_LT_) under different terrain conditions.

**Fig 4:**
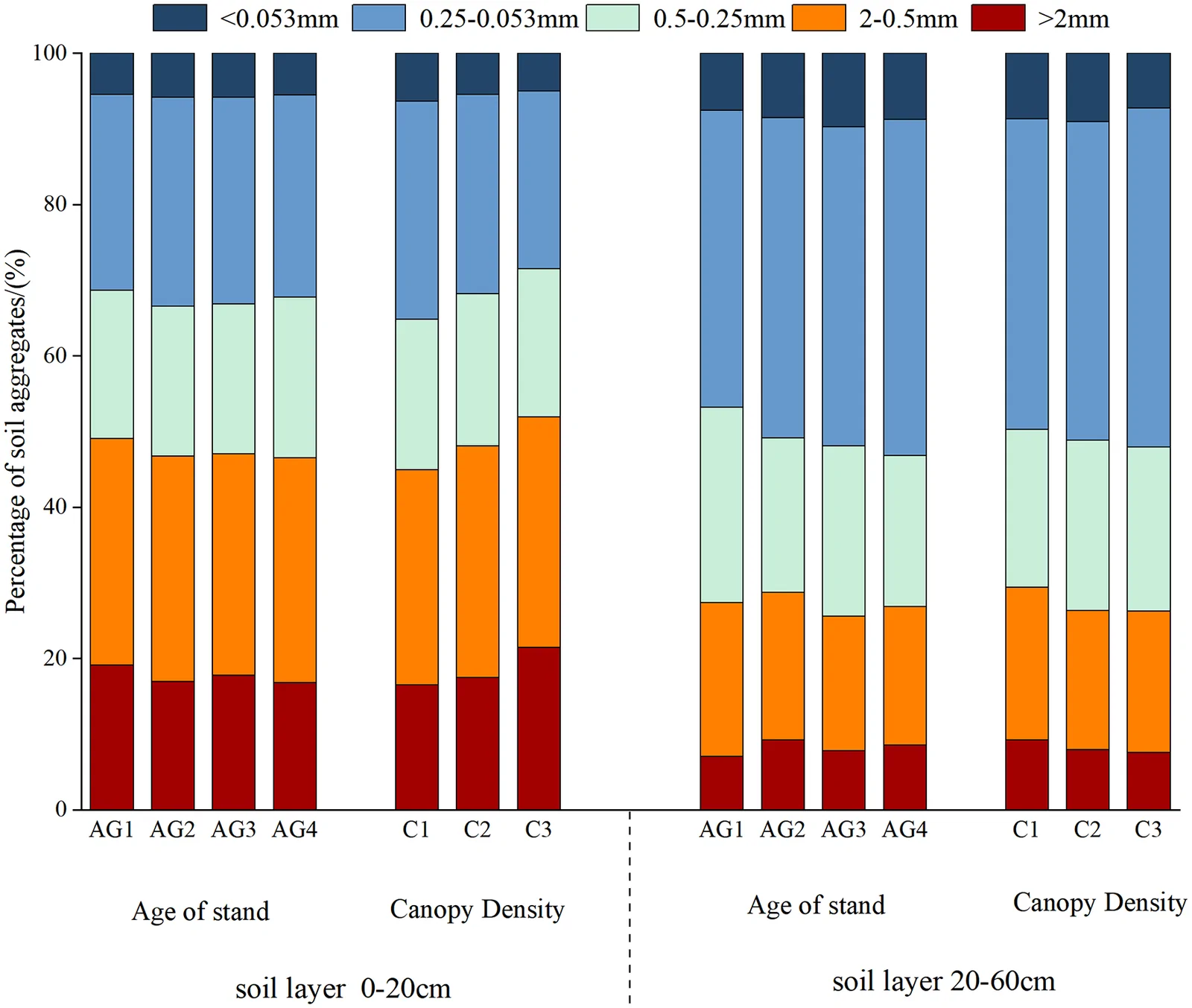
Soil mechanical stability aggregate composition at different depths under different stand conditions. Note: Some letters in the figure represent: AG1: young forest; AG2: middle-aged forest: AG3: near mature forest; AG4: Mature forest; C1: low canopy density; C2: medium canopy density; C3: high canopy.

**Fig 5:**
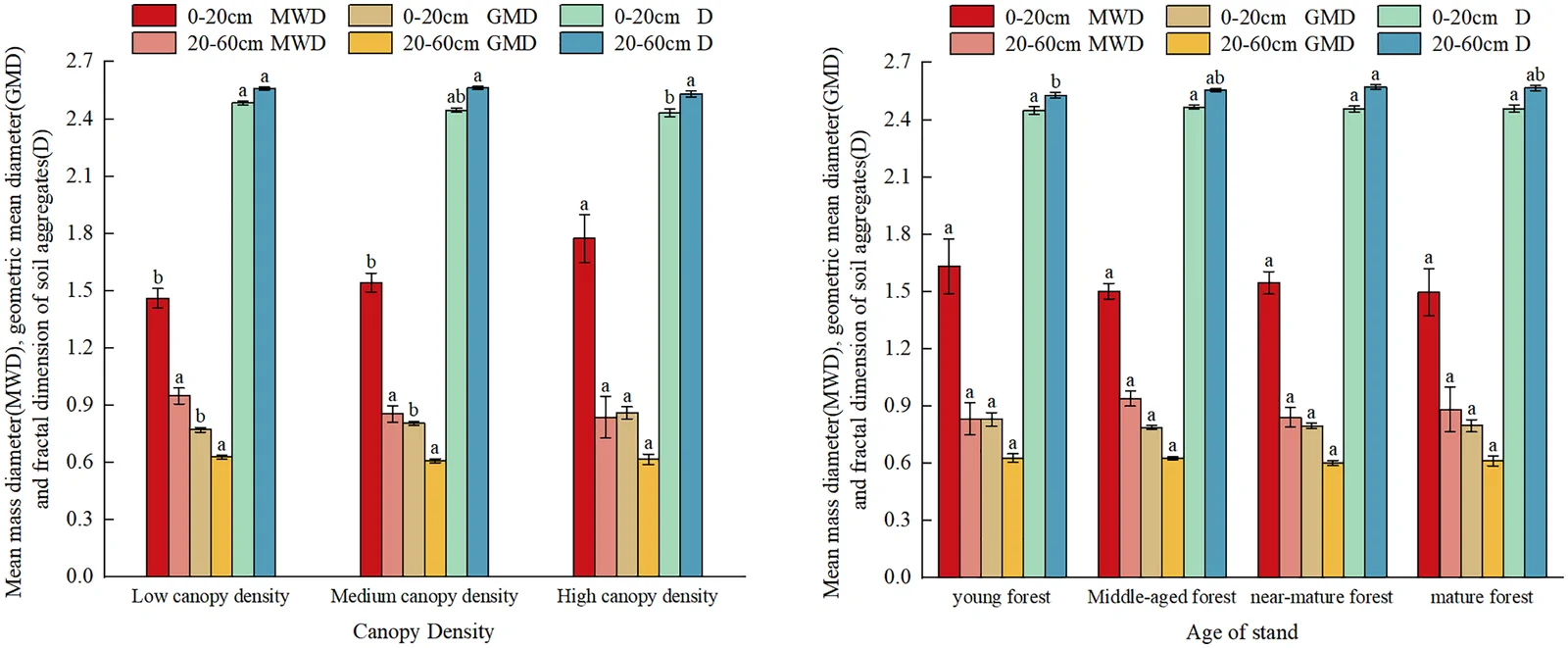
Mean weight diameter (MWD), Geometric mean Diameter (GMD), and fractal dimension (D) of water-stable aggregates under different stand conditions. Note: Different lowercase letters indicate significant differences between different terrain conditions in the same soil layer (P < 0.05).

**Fig 6:**
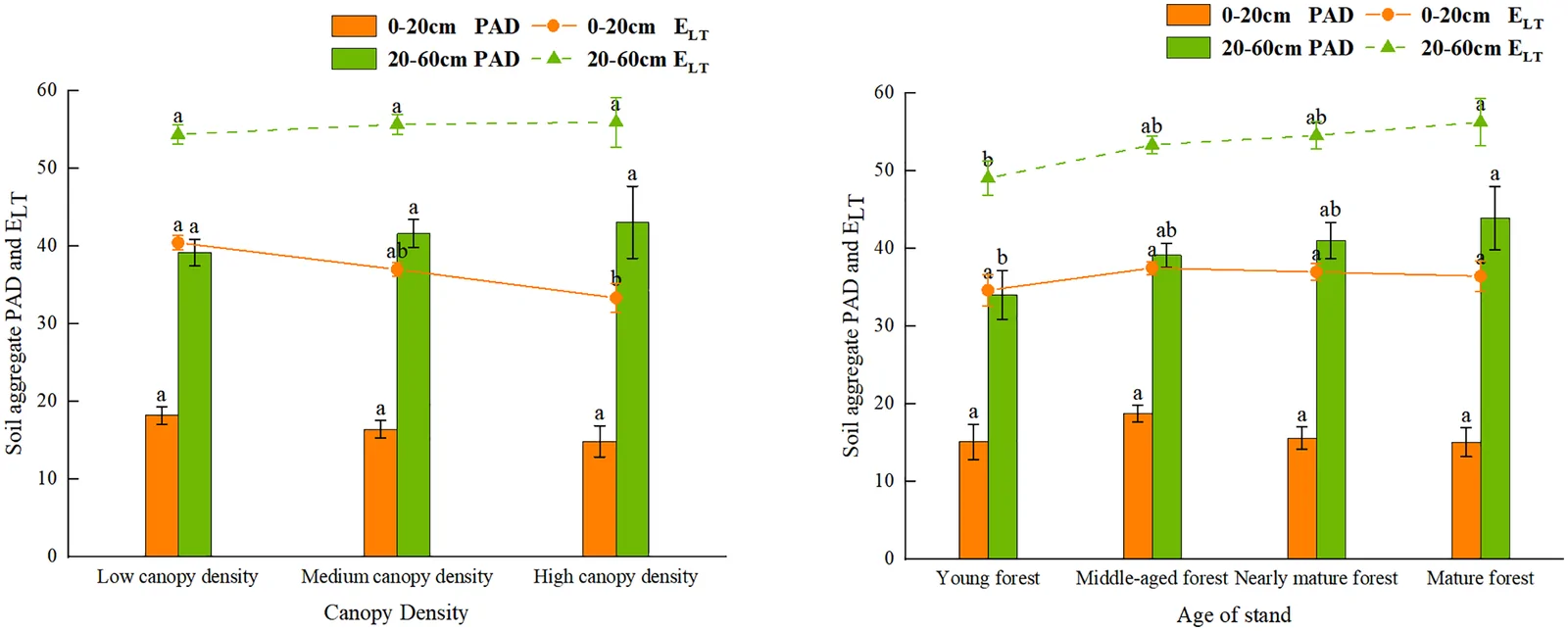
Soil aggregate failure rate (PAD) and unstable aggregate index (E_LT_) under different stand conditions.

To explicitly test hypotheses regarding the relative influence of topography and stand structure on SAS, a multi-tiered analytical framework was implemented:

1. Linear mixed-effects models (MEMs): MWD was the prespecified primary univariate indicator of SAS. The fixed-effects structure included soil depth (0–20 or 20–60 cm), sampling year, altitude, slope gradient, slope position, aspect (exposure), stand age, and canopy closure. Stand age and canopy closure were entered in their measured continuous forms; their categories were used only for graphical summaries. Plot ID was included as a random intercept [MWD ~ depth + year + altitude + slope + position + exposure + stand age + canopy closure + (1 | Plot ID)] to account for the two depth observations originating from the same plot. Sampling year was treated as a fixed blocking factor because only three years were represented and plots were not repeatedly sampled. Models were fitted with the lme4 package using restricted maximum likelihood for coefficient estimation. Fixed-effect estimates were reported with standard errors, denominator degrees of freedom, t statistics, and P values.
2. Redundancy analysis (RDA): RDA was used as an exploratory multivariate complement to the MEM. Whereas the MEM tested adjusted associations with one prespecified response (MWD), RDA summarized how the full set of aggregate-size fractions and, in a separate ordination, the set of stability indices covaried with topographic, stand, and soil predictors. Response and explanatory variables were centered and standardized before analysis. Predictors with variance inflation factors ≥5 were excluded. In CANOCO 5.0, the significance of the constrained ordination was evaluated with 999 Monte Carlo permutations. RDA results were interpreted as multivariate associations and were not used as independent evidence of causal effects.
3. Structural equation modeling (SEM): SEM was used to evaluate a priori, hypothesis-based direct and mediated associations proposed in H3. To limit overfitting, the candidate variables retained after ecological justification and collinearity screening were SOM, SWC, bulk density, slope, exposure, and stand age. The model was fitted in lavaan with maximum-likelihood estimation, and standardized path coefficients were reported. Model adequacy was assessed using the model $\chi^{\text{2}}$ statistic and degrees of freedom, comparative fit index (CFI), and root mean square error of approximation (RMSEA) with its confidence interval. Indirect effects were evaluated with 1,000 bootstrap resamples and 95% confidence intervals. Because the data were observational and cross-sectional at the plot level, arrows represent hypothesized directional associations, not experimentally established causation. The model fit was considered good and acceptable when the following empirical criteria were met: P > 0.05, 1<$\chi^{\text{2}}/\text{df}$<3, RMSEA<0.06, GFI > 0.90.

## Results and analysis

### Spatial patterns of soil aggregate stability across topographic gradients

Water-stable aggregate composition varied across the sampled terrain and stand categories (Table 1; Fig 1). Across categories, the 0.25–0.053-mm fraction was the largest component, whereas the < 0.053-mm fraction was the smallest. Surface soils generally had higher MWD and GMD than subsoils; these patterns are summarized in Table 1 and shown in detail in Figs 1–3.

**Table 1:** Schematic summary of observed patterns in soil aggregate stability (SAS) across topographic and stand factors.

Factor	Surface soil (0–20 cm)	Subsoil (20–60 cm)	Consistent statistical support	Likely mediating mechanisms
**Slope gradient**	MWD slightly higher on 25–35° slopes; PAD increased on steeper slopes; overall moderate effect	Weaker and less consistent patterns	Not significant in MEM	Redistribution of materials; erosion and compaction on steep slopes
**Slope position**	Valley and downhill positions showed relatively higher MWD and lower E_LT_ compared with ridge	Depth-dependent; ridge often showed higher instability	Not significant in MEM	Organic matter accumulation and moisture retention in depositional areas
**Aspect (Exposure)**	Sunny slopes exhibited significantly higher MWD and lower E_LT_ than shady slopes	Similar but weaker	Significant in MEM (p < 0.05)	Radiation-driven differences in soil temperature, moisture, and litter production
**Elevation**	Nonlinear response; 2300–2500 m often showed relatively higher MWD	Nonlinear	Not significant in MEM	Vegetation cover and hydrothermal gradients along elevation
**Stand age**	Younger and middle-aged stands showed relatively higher macroaggregate proportion (R_0.25_); MWD differences moderate	More complex; mature stands sometimes showed slightly higher MWD but also higher instability indices	Significant in MEM (p < 0.05)	Litter input dynamics, root activity, and SOM turnover during forest development
**Canopy closure**	Higher canopy density associated with increased MWD and reduced E_LT_ in surface soil	Depth-dependent; effects weakened in subsoil	Not significant in MEM	Litter retention, reduced evaporation, microclimate stabilization
**Soil organic matter (SOM)**	Strong positive effect on SAS (MWD ↑)	Strong positive	Significant in SEM (β = 0.49**)	Organic binding agents and microbial-derived stabilizers
**Bulk density (BD)**	Strong negative effect (MWD ↓ , PAD ↑)	Strong negative	Significant in SEM (β = −0.41**)	Reduced pore space and root/microbial activity
**Soil water content (SWC)**	Positive indirect effect via SOM; weak negative direct effect	Similar	Significant in SEM	Moisture-mediated regulation of aggregation and SOM dynamics

Descriptively, MWD and GMD tended to be higher on 25–35° slopes, in valley and downslope positions, on sunny aspects, and at 2300–2700 m (Figs 1 and 2). PAD and E_LT_ tended to be higher on slopes >35°, at ridge positions, and on shady aspects (Fig 3). However, in the adjusted MEM, only exposure was a significant topographic predictor of MWD; slope gradient, slope position, and altitude were not significant (Table 2). Thus, category-level patterns for these latter factors should be interpreted as unadjusted trends rather than independent effects.

**Table 2:** A mixed effect model for soil aggregate stability.

Factor group	Estimated value	Standard error	Degrees of freedom	t-value	p-value
**Intercept**	**1.6428**	**0.2333**	**9.724**	**7.041**	**<0.001**
**Altitude**	**−0.0063**	**0.0255**	**376.356**	**−0.245**	**0.806**
**Slope**	**−0.0109**	**0.0293**	**368.700**	**−0.371**	**0.7111**
**Slope Position**	**−0.0185**	**0.0255**	**367.413**	**−0.726**	**0.4682**
**Exposure**	**−0.0905**	**0.0366**	**367.464**	**−2.473**	0.0138*
**Canopy Density**	**0.0409**	**0.0390**	**368.121**	**1.048**	**0.2956**
**Age of stand**	**−0.0763**	**0.0379**	**367.333**	**−2.015**	0.0446*

Note: The significance level is marked as *** P < 0.001, ** P < 0.01, * P < 0.05.

### Effects of forest stand structure on aggregate stability

Aggregate composition also varied among stand-age and canopy-closure categories (Figs 4–6). Younger and middle-aged stands tended to have higher proportions of macroaggregates (R_>0.25_) than mature stands (Fig 4).

Higher canopy closure was descriptively associated with higher surface-soil MWD and GMD and lower E_LT_ (Figs 5 and 6), but canopy closure was not significant after adjustment in the MEM (Table 2). Stand age retained a significant negative coefficient in the adjusted model. In subsoil, stand-category contrasts were weaker and less consistent. Fractal dimension showed little variation among stand categories.

### Bivariate relationships between soil properties and aggregate stability

Bivariate correlations showed that macroaggregate fractions and MWD/GMD were positively associated with SOM, SWC, porosity, and measured nutrients, whereas the < 0.053-mm fraction, PAD, and E_LT_ were positively associated with bulk density and pH (Fig 7). Because these are unadjusted correlations among interrelated soil properties, they indicate covariation and do not establish independent effects.

**Fig 7:**
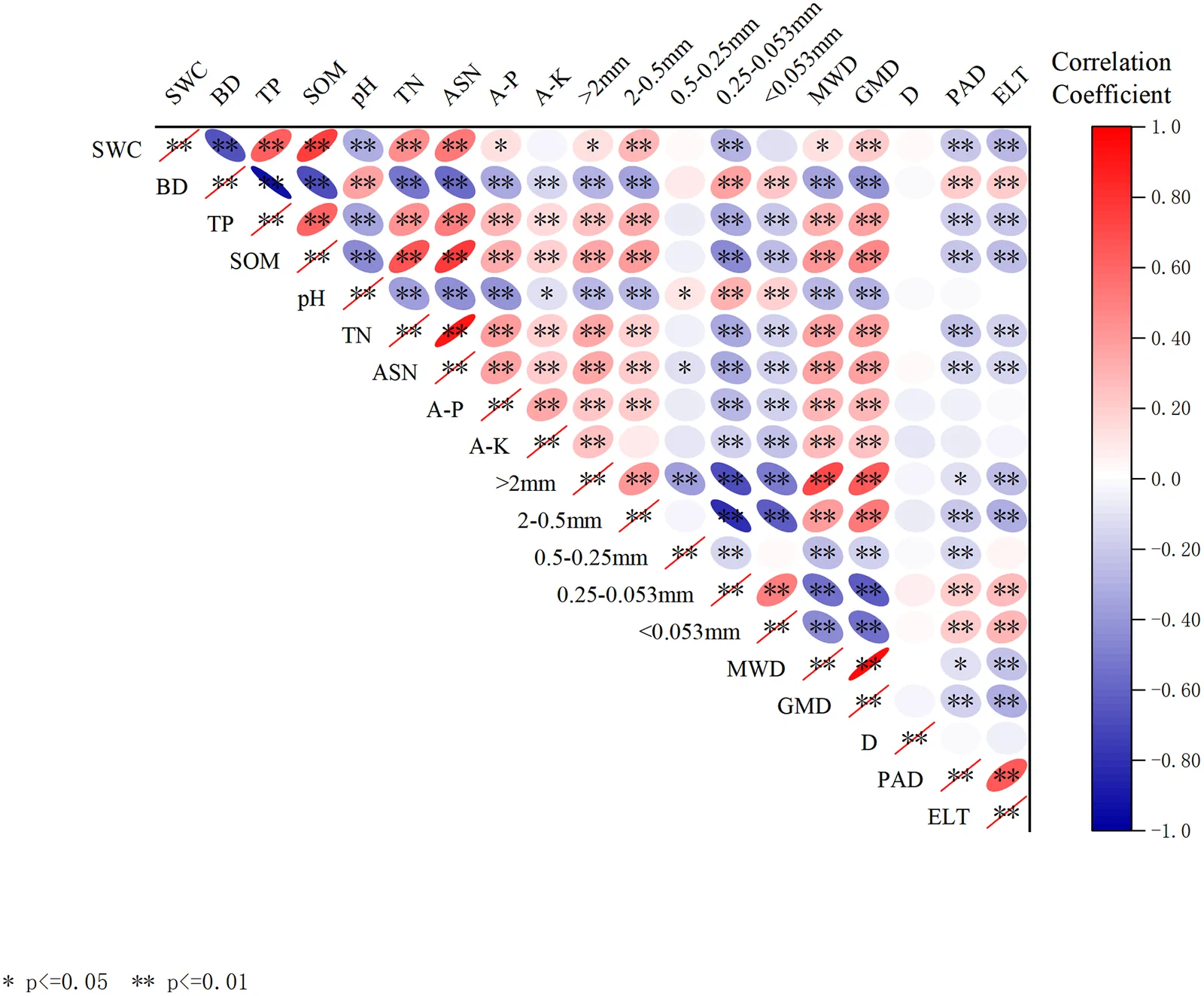
Correlation analysis of soil physicochemical properties and water stability aggregates. Note: ** indicates P < 0.01. * indicates P < 0.05.

### Integrated drivers of soil aggregate stability (MEM, RDA, and SEM)

To explicitly evaluate the relative contributions of topography, stand structure, and soil properties to SAS, an integrated analytical framework (MEM, RDA, and SEM) was applied.

The MEM identified exposure (estimate = −0.0905, P = 0.0138) and stand age (estimate = −0.0763, P = 0.0446) as significant adjusted predictors of MWD (Table 2). Altitude, slope, slope position, and canopy density were not significant (P > 0.05). The coefficient signs depend on the coding of categorical exposure and the scale of continuous stand age; therefore, Table 2 should be read together with the coding definitions in the analysis dataset.

RDA summarized the joint multivariate associations between environmental variables and aggregate responses (Fig 8; Table 3). The first two constrained axes accounted for 18.38% of variation in aggregate composition and 23.23% of variation in the stability-index set. SOM, SWC, and bulk density had the longest vectors in the displayed ordinations. These exploratory patterns complement, but do not override, the adjusted univariate MEM results.

**Table 3:** Summary of Redundancy Analysis (RDA) for soil aggregate composition and stability.

Statistic	Axis 1	Axis 2
**Aggregate Composition (Left Plot)**		
**Eigenvalue**	**0.1616**	**0.0222**
**Variance explained (%)**	**16.16**	**2.22**
**Cumulative variance (%)**	**16.16**	**18.38**
**p-value**	**<0.05**	**<0.05**
**Aggregate Stability (Right Plot)**		
**Eigenvalue**	**0.2163**	**0.0160**
**Variance explained (%)**	**21.63**	**1.60**
**Cumulative variance (%)**	**21.63**	**23.23**
**p-value**	**<0.05**	**<0.05**

Note: Statistical significance of the axes was evaluated using Monte Carlo permutation tests

**Fig 8:**
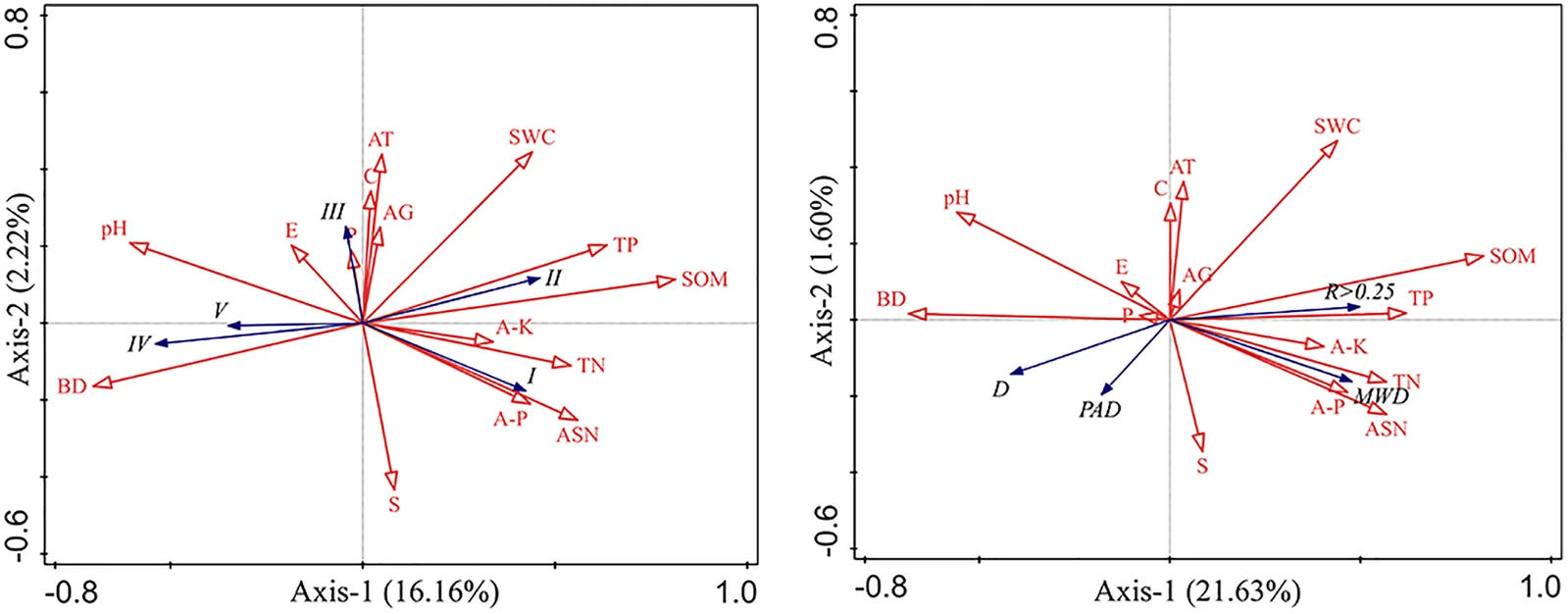
Soil water stability aggregate composition and redundancy analysis of stability and influencing factors (RDA). Note: Some Roman numerals and letters in the figure indicate: S: Slope; P: Slope position; E: Exposure; AT: Altitude; C: Canopy density; AG: Age of stand; I: > 2.0mm; II: 2.0-0.5 mm; III: 0.5-0.25 mm; IV: 0.25-0.053 mm; V: mm; SWC: Water content; BD: bulk density: TP: porosity; SOM: organic matter; TN: total nitrogen; ASN: alkali-hydrolyzed nitrogen; A-P: Available phosphorus; A-K: available potassium.

The SEM was consistent with positive associations of SOM with SAS (standardized β = 0.49, P < 0.01) and SWC with SOM (β = 0.74, P < 0.01), and a negative association of bulk density with SAS (β = −0.41, P < 0.01) (Fig 9). SWC also had a small negative direct path to SAS (β = −0.09, P < 0.01), and slope had a small positive path (β = 0.04, P < 0.01). The model accounted for 23.1% of the variance in SAS. These paths represent associations under the specified model and should not be interpreted as demonstrated causal mechanisms.

**Fig 9:**
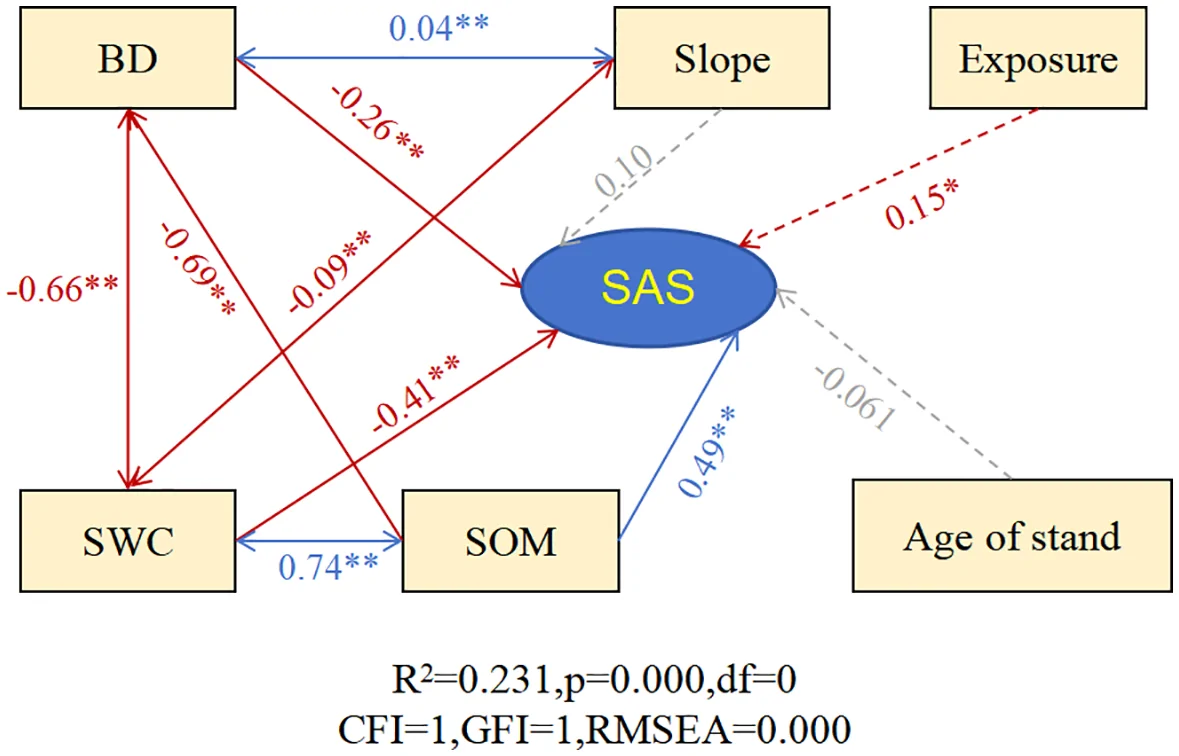
Path Diagram of Structural Equation Model for Factors Influencing Soil Aggregate Stability. Note: Arrows represent hypothesized causal directions, with the numbers next to the arrows indicating the strength of standardized path coefficients. Red arrows indicate positive relationships, while blue arrows denote negative relationships. Only statistically significant relationships are shown (* P < 0.05, ** P < 0.01, *** P < 0.001). The percentage below the ellipses represents the proportion of variance in SAS explained by the model. The following are the variable definitions, SAS: Soil Aggregate Stability; SWC: Soil Water Content; SOM: Soil Organic Matter; Slope: Slope Gradient; Exposure: Slope Aspect; BD: Bulk Density; Age of Stand: Stand Age; Variance Explanation: The percentage below the ellipses indicates the proportion of variance in SAS explained by the model.

## Discussion and conclusions

### Synthesis of findings and hypothesis testing

This study integrated MEM, RDA, and SEM to examine complementary dimensions of SAS variation. H1 received partial support: sunny exposure was associated with MWD in the adjusted MEM, whereas apparent differences among slope, position, and elevation categories did not remain significant after adjustment. H2 also received partial support: stand age was associated with MWD, but canopy closure was not significant in the MEM despite descriptive surface-soil trends. H3 was supported at the level of model-consistent associations: SOM, SWC, and bulk density formed prominent paths between site conditions and SAS, but the observational design does not establish mediation or causation.

### Terrain-mediated mechanisms of soil aggregation

Topography provides a template for the redistribution of water, heat, and soil materials. Valley and moderate-slope plots showed descriptively higher macroaggregate proportions and MWD/GMD, patterns compatible with deposition and retention of moisture and organic matter. Ridge and steep-slope plots tended to show greater PAD and E_LT_. Similar landscape-scale associations have been reported elsewhere [15,16]. Nevertheless, slope and slope position were not significant in the adjusted MEM, so the present data do not demonstrate independent effects of erosion or deposition. Exposure was significant: sunny aspects had greater MWD in the observed coding. Warmer conditions, vegetation production, and root or microbial activity are plausible explanations [22], but these mechanisms were not measured directly.

### Stand dynamics and depth-dependent biological drivers

Stand structure was associated with aggregation, but the evidence differed by predictor. Stand age retained a negative association with MWD in the MEM, while canopy closure did not. Descriptive plots suggested higher surface stability in younger or middle-aged and denser stands. Greater belowground carbon allocation, root turnover, litter retention, and buffering of raindrop impact are plausible mechanisms [23], but root production, litter input, and microbial activity were not quantified in this study.

Stand-category patterns were weaker and less consistent at 20–60 cm than at 0–20 cm. This depth dependence is compatible with stronger litter and root–microbe inputs near the surface and slower organo-mineral processes in subsoil [21]. The observed subsoil patterns in mature stands should be treated as hypotheses for further study rather than evidence of an age-driven shift in carbon allocation.

Within the specified SEM, SOM had the largest positive standardized path to SAS (β = 0.49). This association is consistent with established physical and biochemical roles of organic matter in stabilizing aggregates through roots, fungal hyphae, microbial products, and organo-mineral bonding [24–26]. However, fungal hyphae, root exudates, and microbial necromass were not measured here, so they remain plausible explanations rather than tested mediators.

SWC was positively associated with SOM (β = 0.74) but had a small negative direct path to SAS (β = −0.09). This pattern is compatible with beneficial effects of moisture on plant and microbial processes alongside possible slaking or dispersion under wet conditions [27], although the cross-sectional measurements cannot distinguish those processes. Bulk density had a negative path to SAS (β = −0.41), consistent with reduced pore space and constraints on roots and aerobic activity in compacted soil [28]. These interpretations require experimental or longitudinal confirmation.

### Study limitations and future directions

Several limitations constrain inference. First, the sample represents one experimental forest in the central Tianshan Mountains, so extrapolation to other forest types or climatic regions requires caution. Second, the design was observational; MEM adjusts for measured covariates, while RDA and SEM describe multivariate and hypothesized directional associations, but none establishes causation. Third, the SEM explained 23.1% of SAS variance, leaving substantial variation unaccounted for; global fit indices may also be uninformative if the model is just identified. Fourth, the study did not measure microbial community composition, fungal hyphae, root traits or exudates, litter quantity and chemistry, soil texture/mineralogy, or freeze–thaw and wetting–drying histories. Finally, plots were sampled during different field seasons rather than repeatedly through time, preventing direct assessment of seasonal or interannual dynamics. Long-term repeated measurements combined with microbial, root, mineralogical, and hydrological data are needed to test the proposed mechanisms.

## Conclusions

In the sampled Picea schrenkiana var. tianschanica forest, exposure and stand age were associated with MWD after adjustment for other measured site variables, whereas the apparent effects of slope, slope position, altitude, and canopy closure were not independently significant. RDA and SEM identified SOM, SWC, and bulk density as prominent correlates of aggregate composition and stability. These observational results support management attention to conserving surface organic matter and avoiding compaction, while experimental and repeated-measures studies are needed before specific causal prescriptions can be made.

## Supporting information

S1 Data Data. (XLSX)
